# The BioCyc Metabolic Network Explorer

**DOI:** 10.1186/s12859-021-04132-5

**Published:** 2021-04-21

**Authors:** Suzanne Paley, Peter D. Karp

**Affiliations:** Bioinformatics Research Group, SRI International, 333 Ravenswood Ave, Menlo Park, CA 94025 USA

## Abstract

**Background:**

The Metabolic Network Explorer is a new addition to the BioCyc.org website and the Pathway Tools software suite that supports the interactive exploration of metabolic networks. Any metabolic network visualization tool must by necessity show only a subset of all possible metabolite connections, or the results will be visually overwhelming. Existing tools, even those that purport to show an organism’s full metabolic network, limit the set of displayed connections based on predefined pathways or other preselected criteria. We sought instead to provide a tool that would give the user dynamic control over which connections to follow.

**Results:**

The Metabolic Network Explorer is an easy-to-use, web-based software tool that allows the user to specify a starting metabolite of interest and interactively explore its immediate metabolic neighborhood in either or both directions to any desired depth, letting the user select from the full set of connected reactions. Although, as for other tools, only a small portion of the metabolic network is visible at a time, that portion is selected by the user, based on the full reaction complement, and it is easy to switch among alternate paths of interest. The display is intuitive, customizable, and provides copious links to more detailed information pages.

**Conclusions:**

The Metabolic Network Explorer fills a gap in the set of metabolic network visualization tools and complements other modes of exploration. Its primary strengths are its ease of use, diagrams that are intuitive to biologists, and its integration with the broader corpus of data provided by a BioCyc Pathway/Genome Database.

## Background

Metabolic networks are large and complex graphs that can be daunting to comprehend. Past research has provided multiple modes for exploring metabolic networks. Highly localized modes include viewing single reactions, single metabolites and the reactions they participate in [1, 4, 6], and viewing predefined metabolic pathways, either singly or in connected groupings [1, 3–6, 11]. These approaches lack larger network context. Global modes of exploration include visualization of entire metabolic networks [9, 10, 12], or more precisely, of all metabolites combined with a limited set of reaction edges because including all reactions typically produces an incomprehensible tangle.

The Metabolic Network Explorer fills a gap in the existing modes of exploration, enabling the user to begin at a starting metabolite and interactively explore the metabolic neighborhood around that starting point by moving forward and backward in the network and building up a linear path of arbitrary length. The Explorer enables exploration of connected sets of reactions without the use of pre-defined pathways.

The Metabolic Network Explorer is a new addition to the BioCyc.org website and the Pathway Tools software suite. BioCyc [7] is a collection of Pathway/Genome Databases (PGDBs) for over 18,000 organisms, including EcoCyc, which describes the genes, metabolism, and other functions of *E. coli* K-12 MG1655, and MetaCyc, a database of metabolic reactions and enzymes from all branches of life. The BioCyc website is powered by Pathway Tools [8], a software environment for generating, maintaining, analyzing, visualizing, and web-publishing PGDBs. To access the Metabolic Network Explorer, go to https://BioCyc.org (or any other Pathway Tools website) and select Metabolic Network Explorer from the Metabolism menu. When an organism PGDB is selected, this tool will access the metabolic network of that organism. When MetaCyc is the selected PGDB, this tool accesses metabolites and reactions from all domains of life for applications such as metabolic engineering. Access to EcoCyc and MetaCyc is free; access to other PGDBs beyond a limited number of free monthly pageviews requires a paid subscription. Alternatively, users can install Pathway Tools locally, build a PGDB for any organism of interest from its annotated genome, and use the Metabolic Network Explorer to interactively explore its predicted metabolic network.

We are not aware of other tools that permit the sort of neighborhood-based exploration provided by the Metabolic Network Explorer. Metabolic databases such as BioCyc [7], KEGG [6], Reactome [3], BRENDA [4], and SMPDB [5] provide localized exploration such as visualization of individual reactions and pathways. Tools for visualizing global metabolic networks include offerings from BioCyc [10], KEGG [9], and Reactome [12]. MetExploreViz [2] is a web tool that supports visualization of arbitrary metabolic networks, laid out with a force-directed layout algorithm, but when networks are very large, these diagrams become very difficult to navigate and understand.

## Implementation

The Metabolic Network Explorer is a web application with its display generated entirely using HTML, JavaScript and CSS. The primary data structure is a simple linear path consisting of a list of steps, such that each step consists of a metabolite and an optional successor reaction that links the metabolite to the next step. A second data structure records the metabolic neighborhood of every metabolite that has been retrieved from the database. The metabolic neighborhood of a metabolite *M* is a JavaScript object that includes a near-complete list of possible precursor and successor metabolites based on the set of reactions in the database. (A small number of ubiquitous inorganic molecules, such as water, *H*^+^, and phosphate, are omitted from lists of predecessor and successor metabolites.) A precursor metabolite is any metabolite *P* such that a reaction exists with *P* as a reactant and *M* as a product. A successor metabolite is any metabolite *S* such that a reaction exists with *M* as a reactant and *S* as a product. Some reactions are reversible or of unspecified directionality; in such cases, a metabolite could be either a predecessor or a successor, so is included in both categories. For every neighbor metabolite, we record the list of reactions that link the neighbor to *M*. The data for each reaction includes its full reaction equation, its EC number, the list of enzymes and genes that catalyze the reaction, and any pathways in which the reaction participates.

The user specifies a starting metabolite, and a query is issued to BioCyc or another Pathway Tools server to retrieve the immediate metabolic neighborhood of the selected starting metabolite. The web browser displays the starting metabolite with the list of possible precursors to the left, and the list of possible successors to the right. The user can mouse over any precursor or successor metabolite to see information about the linking reaction(s). The user can also click the plus icon next to any metabolite to add that metabolite to the path. If the metabolite to be added is a precursor to the first metabolite in the path or a successor to the last metabolite in the path, the path is extended in that direction. If the selected metabolite is connected to an intermediate metabolite on the current path, it will replace the existing portion of the path connected to the intermediate metabolite. Thus, only a single linear path (plus all the predecessors and successors for all metabolites on that linear path) can be displayed at a time. When a portion of a path is replaced, the original path is saved to a list of previous paths. The user can easily switch back and forth among previous paths. A path containing more than one metabolite is displayed as a vertical linear pathway diagram, with each reaction optionally including side metabolites, enzymes and genes, EC numbers, compound structures, and links to pathways.

**Fig. 1:**
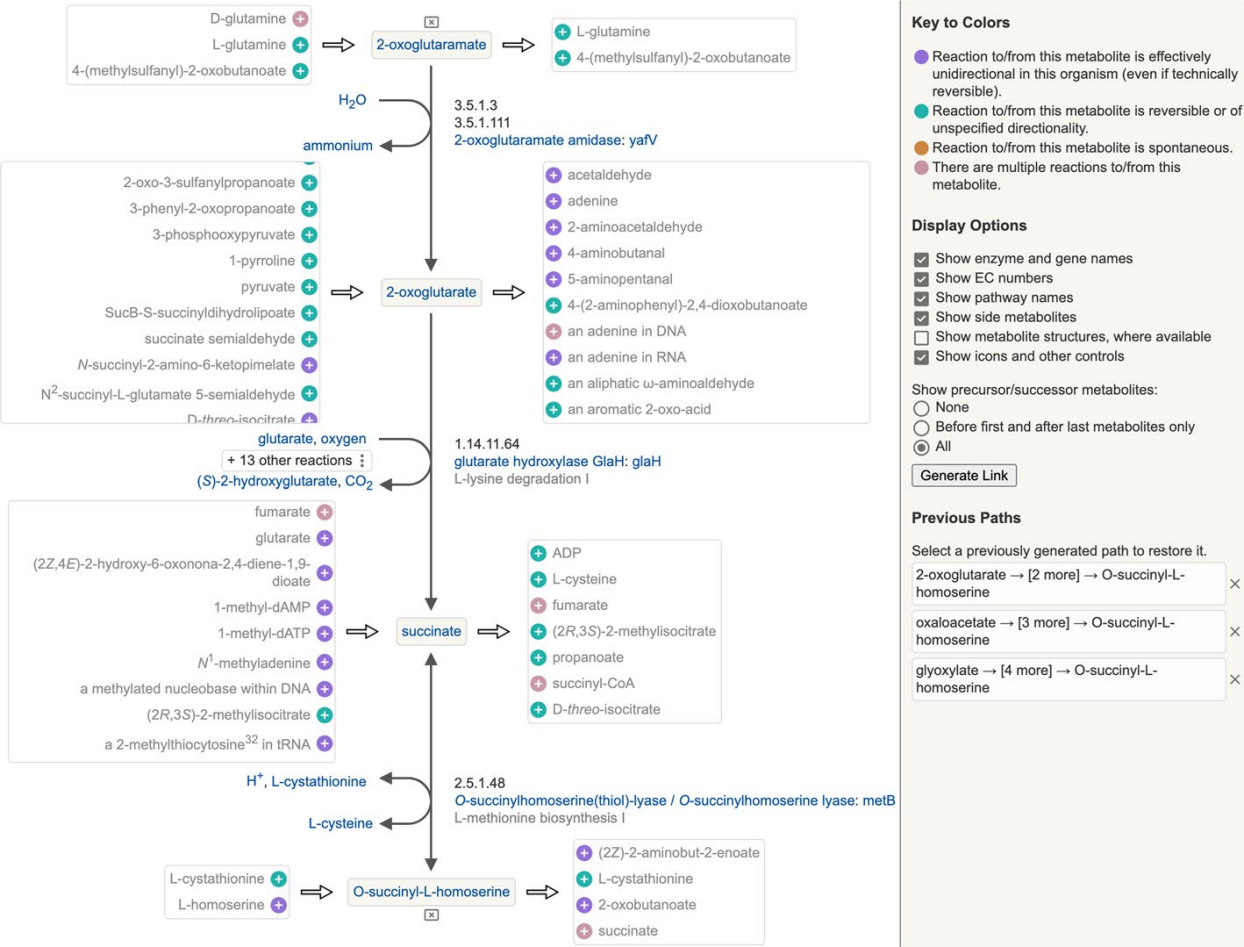
An example Metabolic Network Explorer display, showing an interactively constructed path from 2-oxoglutaramate to O-succinyl-l-homoserine

**Fig. 2:**
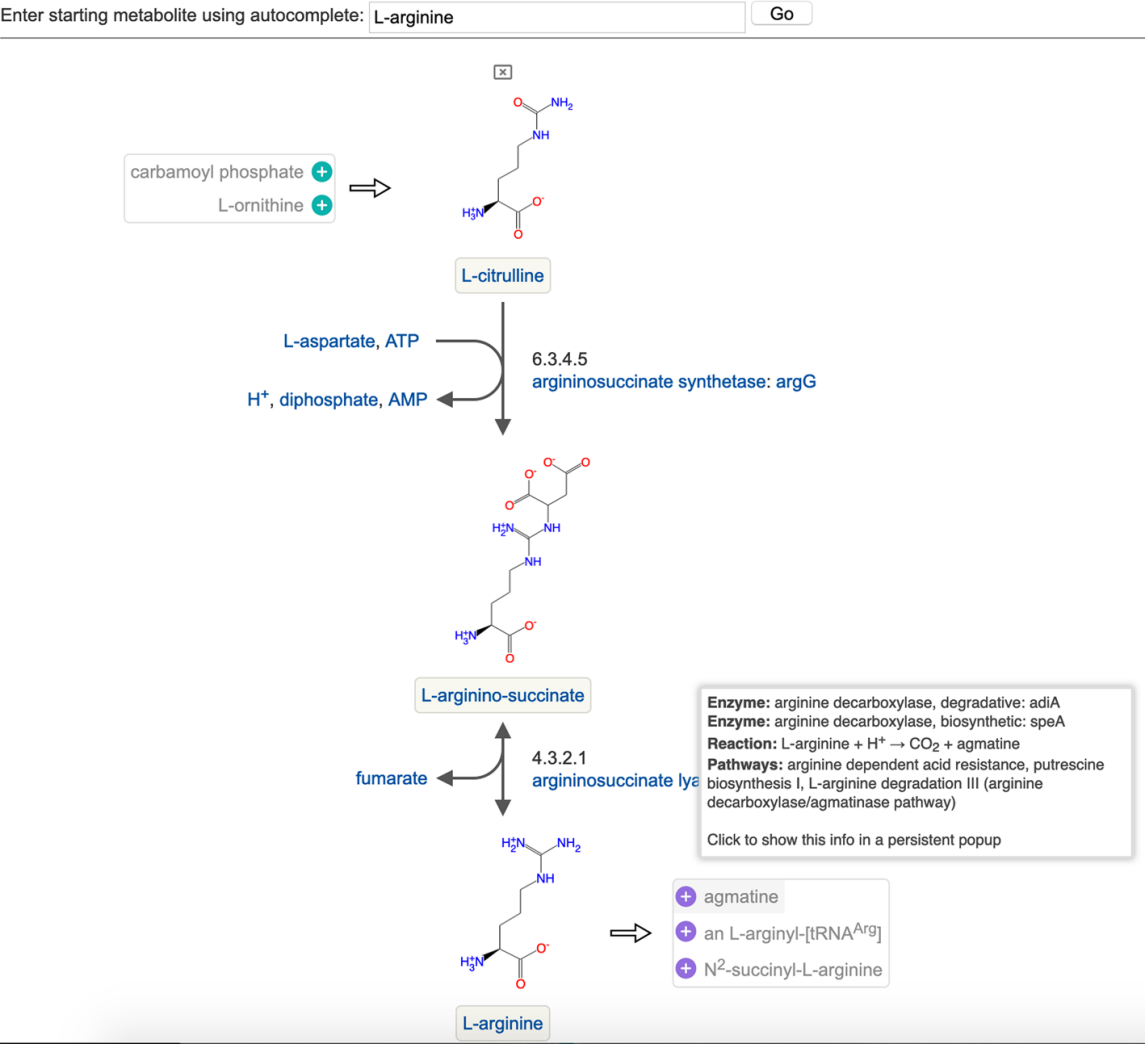
An example Metabolic Network Explorer display, showing a path interactively constructed backwards from l-arginine to l-citrulline. Display of chemical structures has been enabled and display of predecessor and successor candidates for intermediate steps has been suppressed. This display also shows the information presented in the tooltip when the user mouses over a candidate predecessor or successor (in this case agmatine)

## Results

Figure 1 shows a short path that was generated by starting from the metabolite succinate in EcoCyc and interactively expanding backward two steps to 2-oxoglutaramate (top of figure) and forward one step to O-succinyl-l-homoserine (bottom). Other paths were explored along the way; they are listed in the Previous Paths section in the panel at right and can be restored by clicking on them. The display is information-rich, yet easy for a biologist to quickly comprehend. All the potential predecessor or successor metabolites of a given metabolite are collected into a single list (scrollable if the number is large, as is the case for predecessors and successors of oxoglutarate in the figure) displayed to the left or right of each central metabolite, minimizing diagram clutter. Color-coding on the plus icons indicates whether the reaction to and/or from that metabolite is unidirectional, potentially bidirectional, spontaneous, or if there are multiple reactions. When the path includes two metabolites connected by multiple reactions, as in the case of the middle two metabolites in the figure, the number of reactions is listed, and the user can select which is displayed. The diagram is customizable, allowing the user to select which display elements should be visible. Figure 2 shows a second example path in which the display settings have been changed to show compound structures and to suppress display of all predecessor and successor candidates except those at the start and end of the path. Nearly everything in the diagram is clickable. Clicking on a metabolite in the main path, a reaction arrow, or an enzyme, gene or pathway name will open the detailed data page for that object in a separate browser tab. Clicking on a metabolite in the predecessor or successor lists brings up a small panel containing hyperlinks to the metabolite and all reactions, enzymes, genes and pathways that connect to that metabolite, so that the user can examine them in more detail before deciding whether to extend the path to that metabolite. The “Generate Link” button in the right panel generates a URL to the Metabolic Network Explorer preset with the currently displayed path, so that a path can be easily saved or shared.

The result is a tool that enables easy, interactive browsing of a portion of the metabolic network. Unlike other more general network visualization tools, no prior preselection or preloading of a sub-network is required beyond the initial selection of the database of interest. Limiting the tool to showing only a single linear path at a time allows for a compact, easy-to-read, aesthetically appealing display, but full predecessor and successor metabolite lists visible for every metabolite in the selected path make it easy to identify and explore alternate paths. Ready access to detailed information pages for the reactions, pathways and enzymes associated with the current path provides important context.

## Conclusions

The Metabolic Network Explorer is a new web tool that enables interactive generation and display of small metabolic subnetworks of interest without being limited to predefined pathways. Its primary strengths are its ease of use, diagrams that are intuitive to biologists, and its integration with the broader corpus of data provided by a PGDB. It complements other tools for interacting with metabolic networks and fills an important gap in the set of metabolic network visualization tools.

## Data Availability

The data used by this tool are part of the BioCyc data collection, available at https://BioCyc.org. Access to EcoCyc and MetaCyc are free. Access to data for other organisms requires a subscription after a period of free use. The Pathway Tools software is free to academic users with a signed license agreement, and is available for a fee to commercial users. EcoCyc and MetaCyc data are bundled with the software, with data for other organisms requiring a subscription. To obtain the software, see https://BioCyc.org/download.shtml. Source code is available to licensees upon request.

## Abbreviation

PGDB: Pathway/Genome Database
